# Long COVID: The Nature of Thrombotic Sequelae Determines the Necessity of Early Anticoagulation

**DOI:** 10.3389/fcimb.2022.861703

**Published:** 2022-04-05

**Authors:** Chengyue Wang, Chengyuan Yu, Haijiao Jing, Xiaoming Wu, Valerie A. Novakovic, Rujuan Xie, Jialan Shi

**Affiliations:** ^1^ Department of Hematology, The First Hospital of Harbin, Harbin Medical University, Harbin, China; ^2^ Department of Nephrology, The First Hospital of Harbin, Harbin Medical University, Harbin, China; ^3^ Department of Geriatric, Shenzhen People’s Hospital, The Second Clinical Medical College, Jinan University, The First Affiliated Hospital, Southern University of Science and Technology, Shenzhen, China; ^4^ Department of Research, Veterans Affairs (VA) Boston Healthcare System, Harvard Medical School, Boston, MA, United States; ^5^ Department of Medical Oncology, Dana-Farber Cancer Institute, Harvard Medical School, Boston, MA, United States

**Keywords:** long COVID, thrombosis, extracellular vesicles, endothelial injury, chronic hypoxia, inflammation, phosphatidylserine, early anticoagulation

## Abstract

Many discharged COVID-19 patients affected by sequelae experience reduced quality of life leading to an increased burden on the healthcare system, their families and society at large. Possible pathophysiological mechanisms of long COVID include: persistent viral replication, chronic hypoxia and inflammation. Ongoing vascular endothelial damage promotes platelet adhesion and coagulation, resulting in the impairment of various organ functions. Meanwhile, thrombosis will further aggravate vasculitis contributing to further deterioration. Thus, long COVID is essentially a thrombotic sequela. Unfortunately, there is currently no effective treatment for long COVID. This article summarizes the evidence for coagulation abnormalities in long COVID, with a focus on the pathophysiological mechanisms of thrombosis. Extracellular vesicles (EVs) released by various types of cells can carry SARS-CoV-2 through the circulation and attack distant tissues and organs. Furthermore, EVs express tissue factor and phosphatidylserine (PS) which aggravate thrombosis. Given the persistence of the virus, chronic inflammation and endothelial damage are inevitable. Pulmonary structural changes such as hypertension, embolism and fibrosis are common in long COVID. The resulting impaired lung function and chronic hypoxia again aggravates vascular inflammation and coagulation abnormalities. In this article, we also summarize recent research on antithrombotic therapy in COVID-19. There is increasing evidence that early anticoagulation can be effective in improving outcomes. In fact, persistent systemic vascular inflammation and dysfunction caused by thrombosis are key factors driving various complications of long COVID. Early prophylactic anticoagulation can prevent the release of or remove procoagulant substances, thereby protecting the vascular endothelium from damage, reducing thrombotic sequelae, and improving quality of life for long-COVID patients.

## Introduction

Long COVID refers to a long-term multi-system disability syndrome seen in COVID-19 survivors. The US Centers for Disease Control and Prevention (CDC) and National Institutes of Health (NIH) define long COVID as sequelae that extend beyond four weeks after initial infection (Crook et al., 2021). It includes post-acute COVID-19 and post-COVID-19 syndrome. People who have persistent SARS-CoV-2 infection show structural and functional impairment of multiple organ systems, including: respiratory, cardiovascular, haematological, neurological, urinary, gastrointestinal, and musculoskeletal (Sudre et al., 2021). Symptoms include fatigue (47%), dyspnea (32%), myalgia (25%), joint pain (20%), headache (18%), cough (18%), chest pain (15%), olfactory abnormality (14%), taste changes (7%), and/or diarrhea (6%). Heart abnormalities, cognitive impairment, sleep disturbances, post-traumatic stress disorder (PTSD), and concentration problems have also been reported (Aiyegbusi et al., 2021). **Table 1** summarizes other reviews on long COVID symptoms (Ahmad et al., 2021; Cabrerai Martimbianco et al., 2021; Ceban et al., 2021; Groff et al., 2021; Long et al., 2021; Lopez-Leon et al., 2021; Michelen et al., 2021; Alkodaymi et al., 2022; Malik et al., 2022; Nguyen et al., 2022). There have also been recent studies on the pathophysiological mechanism of long COVID. Persistent vascular endothelial injury is common in convalescent COVID-19 patients and is not associated with ongoing acute response (Fogarty et al., 2021). Vascular endothelial damage can be caused by long-term viral infection, chronic hypoxia and inflammatory response. This initiates coagulation and microthrombosis, which may lead to various systemic functional impairments and clinical sequelae (Ambrosino et al., 2021; García-Abellán et al., 2021; Peluso et al., 2021). Thrombosis can further aggravate vasculitis, which may further damage various organs. This is consistent with autopsy findings of coagulation disorders/abnormalities in the lungs and critical organ systems following COVID-19. This also indicates that long COVID is essentially a thrombotic sequela. Unfortunately, there is currently no effective treatment for long COVID. Therefore, more effective early treatment is essential to prevent serious COVID-19 disease, lessen the degree of thrombotic damage, and potentially mitigate long-term sequelae, decreasing the burden of long COVID on patients and healthcare systems.

**Table 1:** Summary of research on persistent symptoms in long COVID.

Reference	Population	Time to assessment	Symptoms (% of patients)
(Groff et al., 2021)	57 studies with 250,351 survivors of COVID-19	1-month after acute COVID-19; 2 and 5 months after infection; 6 months after COVID-19	Generalized anxiety disorder (29.6%); general functional impairments (44.0%); fatigue or muscle weakness (37.5%); difficulty concentrating (23.8%); memory deficits (18.6%), cognitive impairment (17.1%); dysgeusia (11.2%); anosmia (13.4%); headache (8.7%); dyspnea (29.7%); cough (13.1%); mobility decline (20.2%); exercise tolerance (14.7%); joint pain (10.0%); flu-like symptoms (10.3%); general pain (32.4%); persistent fever (0.9%); muscle pain (12.7%); chest pain (13.3%); palpitation (9.3%); gastrointestinal disorders (9.3%).
(Alkodaymi et al., 2022)	63 studies with 257,348 COVID-19 patients	3-<6 months, 6-<9 months, 9-<12 months and ≥12 months	Fatigue, dyspnea, sleep disorder and concentration difficulty (32%, 25%, 24%, and 22% respectively at 3-<6 months follow-up); effort intolerance, fatigue, sleep disorder and dyspnea (45%, 36%, 29% and 25% respectively at 6-<9 months follow-up); fatigue (37%) and dyspnea (21%) at 9-<12 months and fatigue, dyspnea, sleep disorder, myalgia (41%, 31%, 30%, and 22% respectively at >12 months follow-up).
(Ceban et al., 2021)	81 studies	12 or more weeks following COVID-19 infection	Fatigue (32%); cognitive impairment (22%).
(Ahmad et al., 2021)	20 studies	2 weeks to 6 months	The most common prevalent long-term symptoms in COVID-19 patients included persistent fatigue and dyspnea in almost all of the studies. Other reported common symptoms included: shortness of breath, cough, joint pain, chest pain or tightness, headache, loss of smell/taste, sore throat, diarrhea, loss of memory, depression, anxiety.
(Lopez-Leon et al., 2021)	15 studies with 47,910 COVID-19 patients	14 days to 110 days	The five most common symptoms were fatigue (58%), headache (44%), attention disorder (27%), hair loss (25%), and dyspnea (24%). Other symptoms were related to lung disease (cough, chest discomfort, reduced pulmonary diffusing capacity, sleep apnea, and pulmonary fibrosis), cardiovascular (arrhythmias, myocarditis), neurological (dementia, depression, anxiety, attention disorder, obsessive-compulsive disorders), and others were unspecific such as hair loss, tinnitus, and night sweat.
(Cabrerai Martimbianco et al., 2021)	25 studies with 5440 COVID-19 patients	between 3 to 24 weeks after acute phase or hospital discharge	The frequency of long COVID ranged from 4.7 to 80%, and the most prevalent signs/symptoms were chest pain (up to 89%), fatigue (up to 65%), dyspnea (up to 61%), and cough and sputum production (up to 59%).
(Michelen et al., 2021)	39 studies with 10951 COVID-19 patients	12 or more weeks following COVID-19 infection	Weakness (41%); general malaise (33%); fatigue (31%); concentration impairment (26%) and breathlessness (25%); reduced quality of life (37%); reduced pulmonary function (26%)
(Long et al., 2021)	16 studies with 4478 COVID-19 patients	>1 month post-discharge or >2 months post-admission.	Fatigue or weakness (47%); memory impairment (35%); anxiety or depression (33%); dyspnea (33%); hair loss (24%); cardiopulmonary (15%) and neurological system (15%); musculoskeletal system (13%), including myalgia (13%) and joint pain (12%); gastrointestinal symptoms (7%); skin rash (3%); fever (2%).
(Malik et al., 2022)	12 studies with 4828 COVID-19 patients	≥4-weeks post-infection	Fatigue (64%); cough (22.5%); dyspnea (39.5%); anosmia (20%); arthralgia (24.3%), chest pain (10%); headache (21%); sleep disturbances (47%); mental health problems (14.5%).
(Nguyen et al., 2022)	37 studies	≥4 weeks after diagnosis of COVID-19	Fatigue (16-64%); dyspnea (15-61%); cough (2-59%); arthralgia (8-55%); thoracic pain (5-62%).

This article first summarizes the manifestations of abnormal coagulation in long COVID and explains the thrombosis mechanism in detail. Extracellular vesicles (EVs) are released by various cell types to transport cargoes (such as mRNA, microRNAs, DNA, lipids, and various proteins) to nearby or distant cells to help maintain their physiological state. Recent studies have shown that SARS-CoV-2 may be transported by EVs to distant tissues and organs (Barberis et al., 2021; Borowiec et al., 2021; Eymieux et al., 2021). In addition, many studies have shown that EVs play an important role in coagulation activation (Guervilly et al., 2021; Rosell et al., 2021). Long COVID often leads to chronic hypoxia with pulmonary vascular changes and decreased lung function (Carfì et al., 2020; Caruso et al., 2021; Cueto-Robledo et al., 2022). Hypoxia also provides conditions under which immune cells produce more inflammatory cytokines (Moasefi et al., 2021; Østergaard, 2021). Ultimately, prolonged viral presence, hypoxia, and inflammatory responses lead to persistent endothelial damage, extensive vascular endotheliitis and thrombosis. Second, we review and analyze the current studies on the dose and timing of antithrombotic therapy. There is substantial evidence that early anticoagulation therapy improves patient outcomes (Terpos et al., 2020; Arslan et al., 2021; Kollias et al., 2021; Rentsch et al., 2021). In acute COVID-19, the importance of controlling viral replication and preventing inflammation is well established. However, early removal of procoagulant substances and protection of the vascular endothelium may be the best means to prevent long-term thrombotic sequelae.

## Long COVID Coagulation Abnormalities

Several studies have tried to quantify the incidence of ongoing thrombosis in patients after discharge (**Table 2**). Giannis et al. conducted a large-scale (n=4906) statistical analysis of major thromboembolic events in this population. The results showed that 76 patients (1.55%) were diagnosed with venous thromboembolism (VTE), including 44 deep vein thrombosis (0.90%), 42 pulmonary embolism (0.85%), 2 splanchnic vein thrombosis (0.04%), and 3 another vein thromboses (0.06%) (Giannis et al., 2020). Patell et al. showed that the cumulative incidence of thrombosis (including arterial and venous events) at day 30 following discharge was 2.5%; while the cumulative incidence of venous thromboembolism alone at day 30 post discharge was 0.6% (Patell et al., 2020). This shows that discharged COVID-19 patients are still at risk of thrombosis. A study analyzing the serum metabolic profile of 75 previously diagnosed COVID-19 patients 2 months after discharge found that all patients had very high serum concentrations of ferritin and D-Dimer, and 73% had elevated erythrocyte sedimentation rate and CRP (Pasini et al., 2021). Another study showed that plasma samples from Long COVID/PASC (post-acute sequelae of COVID-19) still contain large anomalous (amyloid) deposits (microclots). Various inflammatory molecules were significantly increased in both the supernatant and trapped in the solubilized pellet deposits from Long COVID/PASC samples (Pretorius et al., 2021). A study measuring coagulation indicators 4 months after COVID-19 patients discharge found that the patient samples enhanced thrombin-generating capacity and decreased plasma fibrinolytic potential indicating sustained prothrombotic changes. Increases in plasma factor VIII and PAI-1 levels may be related to the continuous activation of ECs, which may partly explain the hypercoagulable and hypofibrinolytic states (von Meijenfeldt et al., 2021). Korompoki et al. also summarized available evidence on post-acute COVID-19 hematological complications (Korompoki et al., 2022). Overall, these studies have shown that persistent coagulation abnormalities and thrombosis are common in long covid. Other experiments have verified that continuous coagulation activation may lead to abnormal functions in various organs. Post-pulmonary thrombosis syndrome can manifest as persistent thrombosis and long-term functional limitation in long COVID. Pulmonary hypertension, embolism and fibrosis are common sequelae of the lungs (Carfì et al., 2020; Caruso et al., 2021; Cueto-Robledo et al., 2022), which can result in impaired function (diffusing capacity of the lung for carbon monoxide (DLCO), 6-minute walk distances (6MWD), and exercise-induced oxygen saturation) in patients. In summary, the above data indicate that abnormal coagulation is a common manifestation in long COVID, with prolonged coagulation activation, microvascular injury, and thrombosis driving systemic damage in patients.

**Table 2:** Abnormal coagulation in long COVID.

References	Population	Purpose	Results	Conclusions
(Giannis et al., 2020)	N=4906	Postdischarge thromboembolic outcomes and mortality	VTE was diagnosed in 76 patients (1.55%) postdischarge and included 44 DVTs (0.90%), 42 PEs (0.85%), 2 splanchnic vein thrombosis (0.04%), and 3 other vein thromboses (0.06%).	Postdischarge VTE, ATE, and ACM occurred frequently after COVID-19 hospitalization. Postdischarge anticoagulation reduced risk by 46%.
(Patell et al., 2020)	N=163	Postdischarge thrombosis and hemorrhage	The cumulative incidence of thrombosis (including arterial and venous events) at day 30 following discharge was 2.5%; the cumulative incidence of venous thromboembolism alone at day 30 postdischarge was 0.6%.	The rates of thrombosis and hemorrhage appear to be similar following hospital discharge for COVID-19.
(Pasini et al., 2021)	N=75	Serum metabolic profile in pasc syndrome: clinical implication	All patients had very high serum concentrations of ferritin and D-Dimer. 73% had elevations in erythrocyte sedimentation rate and CRP. 27% had elevations in LDH.	The persistence of altered D-Dimer levels raises the possibility of long-term risks of thromboembolic disease.
(Pretorius et al., 2021)	N=49	Investigate whether the persistent symptoms of long-COVID are due to the presence of persistent circulating plasma microclots that are resistant to fibrinolysis.	The plasma samples from long COVID/PASC still contain large anomalous (amyloid) deposits (microclots).	Clotting pathologies in both acute COVID-19 infection and in long COVID/PASC might benefit from following a regime of continued anticlotting therapy to support the fibrinolytic system function.
(von Meijenfeldt et al., 2021)	N=52	Studied the hemostatic status of patients with a resolved COVID-19 infection.	One patient developed a deep vein thrombus with small pulmonary embolisms in the 4 months after hospital discharge. PAI-1 levels were higher in patients compared with controls, both on admission and at 4-month follow-up.	COVID-19 patients have sustained prothrombotic changes as evidenced by enhanced thrombin-generating capacity and decreased plasma fibrinolytic potential at 4 months after hospital discharge.

## Pathophysiology of Long COVID Thrombotic Complications

### Persistence of SARS-CoV-2

Considering the prevalence of the ACE2 receptor, which provides a cellular entry point for SARS-CoV-2, broader organ and tissue damage and long-term complications are not unexpected. A recent study collected blood and nasopharyngeal samples (NPS) to detect SARS-CoV-2 RNA during hospitalization and at 1-, 2-, and 6-months post-discharge. Of 146 patients followed-up, 20.6% required hospital readmission and 5.5% died. SARS-CoV-2 RT-PCR was positive in NPS in 11.8% and 3% of patients at 2 months and 6 months, respectively (García-Abellán et al., 2021). However, whether SARS-CoV-2 can develop into a chronic infection remains to be proven. SARS-CoV-2 infection is known to be associated with accelerated replication and high viral load in the acute phase, with a rapid decline in viral load after the first week (Desimmie et al., 2021). But analysis of autopsy samples from critically ill COVID-19 patients showed viral RNA could be detected before death, suggesting that prolonged virus shedding is associated with serious outcomes (Desimmie et al., 2021).

**Figure 1:**
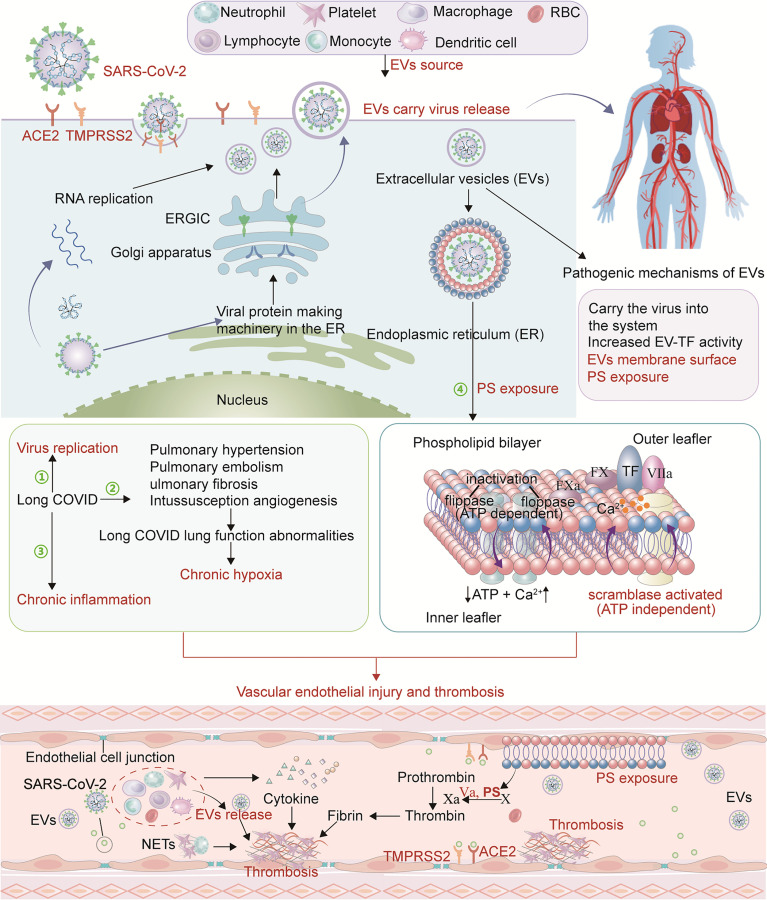
Pathophysiological mechanism of long COVID thrombosis. SARS-CoV-2 enters cells through ACE2 and TMPRSS2 receptors and conducts RNA and protein synthesis and replication. SARS-CoV-2 buds in the ERGIC compartment or Golgi apparatus and exits the cell *via* a biosynthetic secretory pathway. In long-COVID, SARS-CoV-2 may hide in these EVs and re-attack various tissues and organs through the circulatory system. In addition, PS exposure on EVs creates a catalytic surface for clotting factors to facilitate the conversion of prothrombin to thrombin. After cell activation and injury, ATP production is reduced and consumption increases. With the resulting increase in intracellular Ca^2+^, two ATP-dependent transposases (flippase and floppase) are blocked, and ATP-independent scramblases are activated. This leads to the exposure of PS in the outer cell membrane, accompanied by the shedding of microparticles (MPs). PS promotes the decryption of tissue factor (TF) to form TF-FVIIa complex and provides binding sites for procoagulant complexes (endogenous and exogenous fXase and prothrombinase) leading to the generation of thrombin. Pulmonary hypertension, pulmonary embolism and pulmonary fibrosis are common in long COVID resulting in impaired lung function. With the change of lung function, chronic hypoxia inevitably occurs. Hypoxia-induced inflammation may further exacerbate capillary dysfunction and promote thrombosis. Due to SARS-CoV-2 persistence, chronic inflammation in long COVID may be a mechanism that stimulates ECs, platelets and other inflammatory cells, promotes the upregulation of procoagulant factors, and destroys the protective function of vascular endothelium, thereby causing abnormal coagulation.

EVs are lipid bilayer membrane-bound structures released from most eukaryotic cells (such as dendritic cells, neutrophils, monocytes, macrophages, lymphocytes, platelets, mast cells, adipocytes, neurons, epithelial cells and endothelial cells) under physiological and pathological conditions (Yan et al., 2021). EVs contain many biologically active compounds (cargo) such as mRNA, microRNAs, DNA, lipids and various proteins. EVs are classified into three types: exosomes, microparticles (MPs), and apoptotic bodies. Their function is to transport cargo to nearby or distant cells to help maintain their physiological state (Karn et al., 2021). EVs share structural similarities with viruses, such as small size, biogenesis mechanism and cell entry mechanism, etc. (Borowiec et al., 2021). Most enveloped RNA viruses are released by budding from the plasma membrane or by budding within the host cell. The same SARS-CoV-2 buds in the ER–Golgi intermediate compartment (ERGIC) or Golgi apparatus can enter the extracellular space *via* the biosynthetic secretory pathway (Eymieux et al., 2021). Research suggests that SARS-CoV-2 has the potential to leave cells as small secretory vesicles that then release virus (Eymieux et al., 2021). Another study found the presence of SARS-CoV-2 RNA in exosomal cargo, suggesting that the virus may transmit infection through the endocytic pathway (Barberis et al., 2021). This suggests that the cellular transport pathway associated with the release of EVs carrying SARS-CoV-2 may be one of the potential mechanisms for recurrence of COVID-19 infection. EVs may play a ‘Trojan horse’ role in viral RNA reappearance in recovered COVID-19 patients (Borowiec et al., 2021). In long-COVID, SARS-CoV-2 may hide in these EVs and re-attack various tissues and organs through the circulatory system (**Figure 1**).

In addition to their function as transporters, EVs play an important role in inflammation, coagulation, and immune regulation. Studies have shown that EV-TF activity is significantly increased in hospitalized patients with COVID-19, and TF-positive EVs are released into the circulation, which may lead to thrombosis, increasing disease severity and mortality (Guervilly et al., 2021; Rosell et al., 2021). Phosphatidylserine (PS) is a membranous phospholipid normally sequestered in the inner leaflet of a cell membrane. When vascular ECs and circulating blood cells are damaged, the flippases and floppases that maintain the asymmetric lipid distribution in the membrane are blocked, and scramblase is activated. This leads to PS exposure in the outer cell membrane, accompanied by the shedding of MPs (Bevers and Williamson, 2016). PS exposure in the outer leaf of the cell membrane due to viral infection may be another mechanism of acute immune-inflammatory response and coagulation activation (Argañaraz et al., 2020). PS exposure creates a catalytic surface for clotting factors which facilitate the conversion of prothrombin to thrombin (**Figure 1**). PS-exposing, sub-micron sized EVs, termed microparticles (MPs), have been shown to have important effects on coagulation. Indeed, COVID-19 patients exhibit an accumulation of TMPs (total MPs), PMPs (platelet MPs), EMPs (ECs MPs), and activated platelets (Zahran et al., 2021). Another study showed that platelet PS externalization in COVID-19 patients is associated with increased D-dimer. Compared with patients without thrombosis, patients with thrombosis had significantly higher PS externalization (Althaus et al., 2021). The above studies lead us to speculate that EVs can carry the virus to reach distant tissues and various organs including the vascular system, and re-injure the vascular endothelium and systemic system. The expression of tissue factor and PS exposure on the EVs surface are also important factors in promoting coagulation disorders. These may all be mechanisms to explain the complications in long-COVID patients.

### Chronic Hypoxia and Persistent Immune Disorders

As previously mentioned, pulmonary hypertension, pulmonary embolism and pulmonary fibrosis are common in long COVID resulting in impaired lung function. Autopsy results have shown severe changes in COVID-19 lung structure, with loose alveolar membrane fibrin network and fibrinohemorrhagic alveolitis. Pulmonary vascular changes were evident, including extensive endothelial damage and thrombosis. Fibrous microthrombi are frequently found in alveolar septal capillaries. Furthermore, capillary hyperplasia is frequently detected in the alveolar septum, suggesting intussusception angiogenesis (IA) (Congiu et al., 2021). These vessels cause severe distortion of the alveolar-capillary plexus structure. Unlike ARDS patients, the degree of pulmonary vascular shunting in COVID-19 patients is associated with poor blood oxygenation (Østergaard, 2021). With these changes in lung function, chronic hypoxia inevitably occurs (**Figure 1**), leading to conditions under which immune cells produce more inflammatory cytokines (Moasefi et al., 2021). Hypoxia-induced inflammation may further exacerbate capillary dysfunction, creating a vicious cycle. Hypoxia can activate the transcription factor early growth response-1, upregulate tissue factor expression in mononuclear phagocytes, and promote changes in the fibrinolytic system, such as increased expression of plasminogen activator inhibitor-1 (PAI-1), thereby promoting thrombosis (Gupta et al., 2019; Thachil, 2021). Furthermore, hypoxia leads to activation and apoptosis of endothelial cells (ECs), reducing their anticoagulant properties and enhancing vascular permeability, leukocyte adhesion, and MPs production (Deng et al., 2018; Evans, 2019). Importantly, hypoxemia-induced thrombosis can lead to increased metabolic toxins, energy deficit, extensive cellular damage and death, and multiple organ failure.

Cytokine storm can exacerbate the severity of acute COVID-19 in hospitalized patients. However, replication-competent viruses are rarely recovered beyond 20 days after symptom onset, suggesting that persistent symptoms may be driven by an immune response (Amenta et al., 2020). Peluso et al. demonstrated that during early recovery, those who went on to develop PASC generally had higher levels of cytokine biomarkers including TNF-α, IFN-γ–induced protein 10 and IL-6 (Peluso et al., 2021), consistent with increased immune activation. Some speculate that persistent viral RNA shedding triggers chronic immune activation (Desimmie et al., 2021; Fernández-Lázaro et al., 2021). Immune system dysregulation in long COVID is characterized by increased interferon gamma (IFN-γ) and interleukin (IL)-2, pathological changes in CD4^+^, CD8^+^ lymphocyte subsets, monocyte CD14^+^ and CD16^+^ subsets, and defects in B lymphocytes and monocytes. Increased oxidative phosphorylation and reactive oxygen species-related inflammatory responses displace TNF-α and IL-6-driven inflammatory responses, driving persistent symptoms and progression of long COVID (Fernández-Lázaro et al., 2021). Thus chronic persistent inflammation in long COVID may stimulate ECs, platelets and other inflammatory cells, promote the upregulation of procoagulant factors, and destroy the protective function of vascular endothelium, thereby causing abnormal coagulation (**Figure 1** **)**. These effects create a feedback loop where inflammation causes thrombosis, and the resulting blood clots can directly contribute to inflammation. Thrombin cleaves fibrinogen and activates the cytokine IL-1α, providing a direct link between coagulation and inflammation (Stark and Massberg, 2021).

Autoantibodies that promote thrombosis have long been recognized as an important factor in COVID-19 progression (Knight et al., 2021). Antiphospholipid autoantibodies (APL), in particular, promote thrombosis both by stimulating neutrophils to release neutrophil extracellular traps and by activating ECs and platelets (Chen et al., 2021; Knight et al., 2021). However, it is unclear how long autoantibodies will persist, and their role in long COVID remains to be studied.

### Endothelial Damage and Persistent Dysfunction

**Figure 2:**
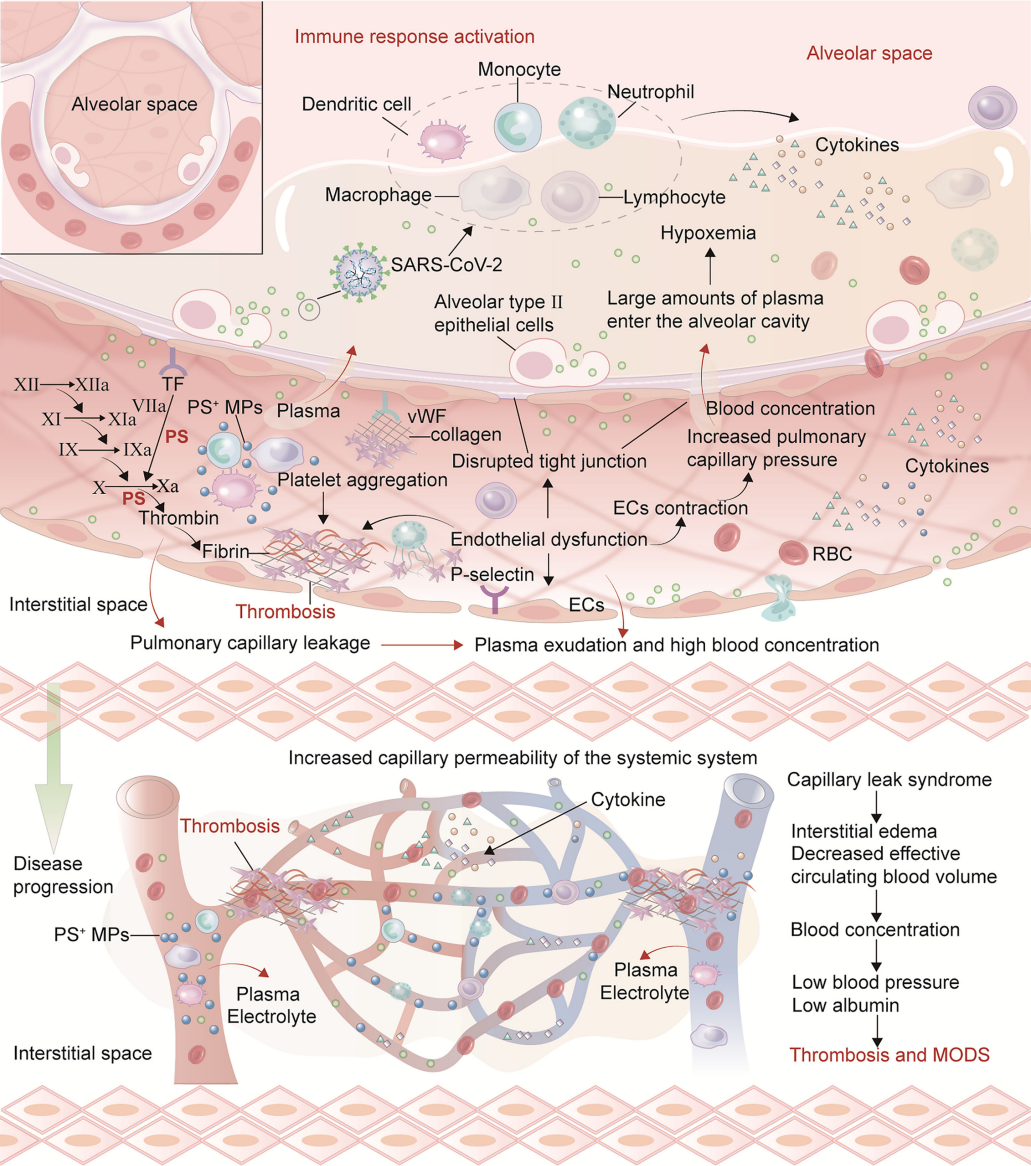
Mechanisms of endothelial injury promoting thrombosis and CLS in acute COVID-19 and long COVID. After vascular endothelial injury, there may be weakened anticoagulant properties, increased permeability and leukocyte adhesion. TF expression on ECs surface is up-regulated. Antithrombin III, TF pathway inhibitor and protein C system are damaged and lose anticoagulant properties. Injured ECs can release vWF, factor VIII and PS exposure to promote a hypercoagulable state. Furthermore, ECs can increase the expression of chemokines on their surface, promote neutrophil recruitment, and participate in thrombosis. SARS-CoV-2 and cytokines (such as TNF-α, IL-1, IL-6) damage the vascular endothelium, resulting in ECs contraction, connections separating and the appearance of intracellular gaps. The general increase in capillary permeability forms a local or SCLS. The increased permeability of pulmonary capillary endothelial injury can lead to plasma entering the alveolar cavity and form hypoxemia. Furthermore, hypoxia aggravates the contraction of pulmonary capillary ECs which thicken and narrow the capillaries, ultimately causing pulmonary hypertension. The plasma and some erythrocytes in the pulmonary capillaries are pushed into the alveolar space, further aggravating respiratory dysfunction and ARDS. As the disease progresses, injury to circulating blood cells and vascular endothelium can activate cytokines release, resulting in extensive capillary ECs damage, increasing the transport channel diameter and vessels permeability, and albumin leakage in the blood vessels.

In multivariate analysis, endothelial dysfunction is an independent risk factor for long COVID syndrome (Charfeddine et al., 2021). Vascular endothelial injury is also common in long COVID. EC biomarkers including vWF: Ag, vWF propeptide (vWFpp) and Factor VIII (FVIII: C) are significantly elevated in convalescent COVID-19 patients (Fogarty et al., 2021). Another study has shown that post-acute COVID-19 syndrome is associated with persistent and sex-biased endothelial dysfunction, directly related to the severity of pulmonary impairment (Ambrosino et al., 2021). Vascular endothelial injury is the central link between the mechanisms that promote thrombosis. ECs cover the entire vascular system, regulate blood flow and coagulation, initiate and amplify inflammation, and maintain vascular tension, structure and homeostasis (Rodríguez et al., 2021). Autopsy studies have shown that SARS-CoV-2 infection has a wide range of serious effects on ECs, including (but not limited to) severe endothelial injury and endotheliitis, capillary inflammation, extensive microvascular disease, thrombosis and new abnormal angiogenesis (Levi and Coppens, 2021). Vascular endothelial injury increases permeability and leukocyte adhesion while weakening the cells’ anticoagulant properties through decreases in antithrombin III, tissue factor pathway inhibitor and protein C. Injured ECs become procoagulant by upregulating tissue factor (TF) expression, exposing PS, and releasing vWF and factor VIII. Furthermore, ECs can increase the expression of chemokines on their surface, promote neutrophils recruitment, and participate in thrombosis (Birnhuber et al., 2021; Levi and Coppens, 2021) (**Figure 2** **)**. ECs disorders caused by inflammation may lead to a massive increase in plasminogen activator, consistent with the high D-dimer levels in severe COVID-19 patients. Also, plasmin effects on metalloproteinases can cause extracellular matrix modification and accelerate capillary leakage. Therefore, endothelial injury and persistent dysfunction may also play a role in post-acute symptoms and organ dysfunction (Flaumenhaft et al., 2022).

Under physiological conditions, blood is a viscous fluid that will form a coaxial fluid layer in the blood vessels. Due to friction with the blood vessel wall, the blood divides into many layers with sequentially decreasing flow rates from inside to outside. The high shear stress found in laminar flow is optimal for EC survival and quiescence, promoting vasodilation and the flow and secretion of anticoagulant substances. Low or changing shear stress in turbulent flow leads to EC proliferation, deformation, and apoptosis, promoting vasoconstriction, coagulation, and secretion of platelet aggregation substances (Styp-Rekowska et al., 2011). In COVID-19, damage to the endothelium by virus, inflammation, and hypoxia may reduce flow rate and wall shear stress, prompting platelet aggregation and thrombosis (Ackermann et al., 2020a). Furthermore, intussusception angiogenesis (IA) is one of the manifestations of endothelial dysfunction that is observed in various organs in deceased COVID-19 patients. This is a rapid angiogenesis process that splits the blood vessel into two lumens by the incorporation of circulating angiogenic cells (Ackermann et al., 2020a; Ackermann et al., 2020b). Hypoxia, classical angiogenic molecular factors, excessive inflammation and cytokine storm, thrombosis, related hemodynamic changes, and dysregulation of RAAS products are all important factors contributing to IA (Madureira and Soares, 2021). The vascular regulation disorder in focal vasoconstriction and progressively dilated vessel segments may also interfere with physiologic laminar flow (Ackermann et al., 2020a).

**Figure 3:**
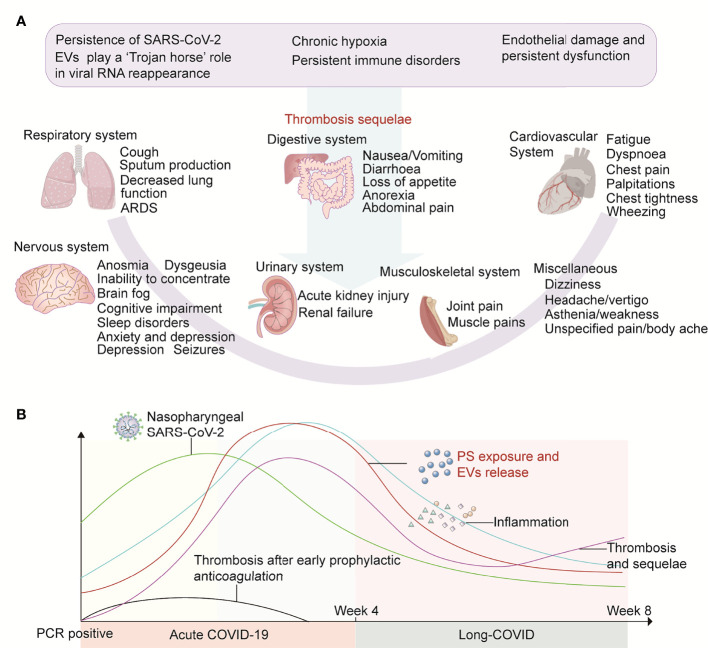
Thrombotic sequelae and possible outcomes of early anticoagulation in long COVID. **(A)** In long COVID, EV-delivered virus persistently attacks systemic systems, coupled with chronic hypoxia and persistent inflammatory responses, which collectively damage the vascular endothelium. The above factors also lead to PS exposure on the surface of various types of cells and EVs from which they are derived. These factors influence each other and together promote thrombosis. **(B)** We propose a hypothesis that early prophylactic anticoagulation in COVID-19 can quickly remove a variety of procoagulant substances, thereby protecting the blood system and surrounding tissues and organs from damage, inhibiting PS exposure to initiate coagulation, and avoiding thrombosis and sequelae.

Studies have reported acute respiratory failure caused by pulmonary capillary leak syndrome (CLS) after SARS-CoV-2 infection (Bahloul et al., 2021). A study showed that in mild to moderate COVID-19, patients with known or suspected systemic capillary leak syndrome (SCLS) may have an increased risk of disease emergencies (Cheung et al., 2021). Under normal physiological conditions, water and electrolytes can enter the interstitial space through the capillary barrier due to changes in the osmotic balance, while substances with slightly larger molecular masses such as albumin cannot. In the early stage, SARS-CoV-2 replication initiates innate and acquired immune responses, promotes immune cells recruitment, releases cytokines, and leads to cell damage and death. Viruses and cytokines (such as TNF-α, IL-1, IL-6) damage the vascular endothelium, resulting in ECs contraction (Bahloul et al., 2021; Lacout et al., 2021). The general increase in capillary permeability forms a local or systemic CLS. This increased permeability can lead to plasma entering the alveolar cavity, resulting in hypoxemia. Furthermore, hypoxia aggravates the contraction of pulmonary capillary ECs which thicken and narrow the capillaries, causing pulmonary hypertension. Plasma and erythrocytes from pulmonary capillaries are pushed into the alveolar space, further aggravating respiratory dysfunction and ARDS. As the disease progresses, injured circulating blood cells and vascular endothelium can release cytokines, resulting in extensive capillary ECs damage, increasing the transport channel diameter, vessels permeability, and albumin leakage (**Figure 2** **)**. Patients can have the typical features of CLS: low volume hypotension, hypoalbuminemia and hemoconcentration triad with systemic edema. In severe cases, multiple organ dysfunction syndrome (MODS) may occur, affecting heart, lung, and kidneys. Concentration and obstruction of blood aggravates the accumulation of procoagulant substances. ECs contraction causes capillaries stenosis, which makes it easier for blood components to accumulate and produce further EC damage. Although there has yet to be a report of confirmed CLS in long COVID, abnormal endothelial function and thrombosis will hinder the patient’s long-term recovery, aggravating symptoms and system dysfunction. In conclusion, long-COVID pathogenesis may be explained by the combined effects of chronic hypoxia, persistent inflammatory response, and thrombosis on vascular ECs. Understanding the mechanism of coagulation abnormalities in the long COVID can help inhibit thrombosis more effectively and prevent disease progression and sequelae (**Figure 3A** **)**.

## Trends in Early Anticoagulation in COVID

Anticoagulation is a common treatment for hospitalized patients with COVID-19. Many articles have discussed the optimal dosing and duration of anticoagulant treatment (Jonmarker et al., 2020; Taccone et al., 2020; Atallah et al., 2021; Wahid and Ortel, 2021; Bradbury and McQuilten, 2022). It is certain that thrombosis risk gradually increases with disease progression, necessitating the use of anticoagulants (such as heparin, LMWH and DOAC) in the middle and late stages to inactivated coagulation factors and inhibit re-formation of thrombi. Yet its effects in these patients does not depend on increasing dose. This is likely because the anticoagulants cannot completely remove the large number of thrombi in patients with advanced disease. In contrast, early application of anticoagulants in COVID-19 has shown beneficial results (Terpos et al., 2020; Arslan et al., 2021; Kollias et al., 2021; Rentsch et al., 2021). Arslan et al. found that patients who received LMWH had shorter hospital stays compared with those who did not receive LMWH despite being older, with more comorbidities (such as hypertension, coronary heart disease and cancer) and higher inflammatory markers (C-reactive protein). Early anticoagulation in this study refers to the treatment of patients without any contraindications in early stage COVID-19 infection. We can speculate that early anticoagulation therapy would benefit more patients with advanced age, more underlying comorbidities, and higher inflammatory markers. Another study also found that starting prophylactic anticoagulation within 24 hours of admission reduced 30-day mortality and in-hospital mortality. Evidence of benefit is strongest in patients not admitted to the ICU within 24 hours of admission (Rentsch et al., 2021). Sulodexide is a compound of two glycosaminoglycans (GAGs): a fast-moving heparin fraction (80%) and dermatan sulfate (20%). In addition to being effective in anticoagulation, sulodexide also restores endothelial barrier function. Sulodexide has a lower risk of bleeding than other oral anticoagulants. A study of early outpatient patients with mild COVID-19 has shown that sulodexide is effective in reducing hospitalizations and the need for supplemental oxygen therapy. Patients treated with sulodexide also had lower CRP and D-dimer, inflammation markers and pre-thrombotic status (Gonzalez-Ochoa et al., 2021). In conclusion, the above data provide high-quality evidence for early anticoagulation in COVID-19. Given that early prophylactic anticoagulation in COVID-19 is a new treatment trend, more research is needed to explore which group of patients will benefit the most and determine the duration of treatment.

In **Figure 3B** of this paper, we propose a hypothesis that in long COVID, EV-delivered virus persistently attacks the systemic system, and coupled with chronic hypoxia and persistent inflammatory response, damages the vascular endothelium. The above factors also lead to PS exposure on the surface of various cells and their derived EVs. These factors influence each other and together promote thrombosis. Early prophylactic anticoagulation in COVID-19 can quickly remove a variety of procoagulant substances, thereby protecting the blood system, surrounding tissues, and organs from damage, inhibiting PS exposure, and avoiding subsequent thrombosis and sequelae (**Figure 3B** **)**.

Some studies suggest that extending venous thromboembolism prophylaxis beyond hospital discharge may be beneficial, but the benefit is limited to high-risk patients with an increased risk of thromboembolism from COVID-19. For example, in one study analyzing 146 patients, 28% were prescribed post-discharge thromboprophylaxis. Its results suggest greater use in patients with higher levels of maximal D-dimer and C-reactive protein after and during ICU admission. Strategies to selectively provide thromboprophylaxis appear to be safe and potentially effective in high-risk patients (Engelen et al., 2021). Another study in high-risk patients (increased risk of venous thromboembolism) hospitalized and discharged for COVID-19 showed that 35 days of rivaroxaban with thromboprophylaxis improved clinical outcomes compared with unextended thromboprophylaxis (Ramacciotti et al., 2022). However, one study reported a low rate of vascular thromboembolic events after discharge in patients with COVID-19 and suggested that thromboprophylaxis should not be routinely used in patients with COVID-19 after discharge (Eswaran et al., 2021). The authors speculate that it is possible that patients at higher risk for vascular thromboembolic events (VaTEs) were on prophylactic anticoagulation at discharge, which may have contributed to the lower rates in the VaTEs group. Many guidelines also recommend the use of anticoagulants after discharge (Barnes et al., 2020; Bikdeli et al., 2020; Moores et al., 2020; Spyropoulos et al., 2020; Vanassche et al., 2020; Cuker et al., 2022). ASH recently issued a conditional recommendation not to use outpatient anticoagulation prophylaxis for discharged COVID-19 patients without suspected or confirmed venous thromboembolism or other indications for anticoagulation (Cuker et al., 2022). The panel judged that both the benefits and harms of thromboprophylaxis after discharge were negligible in absolute terms. Despite a small benefit and reduction in mortality from venous thromboembolism with anticoagulation after discharge, the certainty of the evidence is low. Meanwhile, in COVID-19, there is no high-quality direct evidence that anticoagulation increases the risk of major bleeding complications. However, the panel believes that for patients without COVID-19, there is high-quality indirect evidence that there is an increased risk of major bleeding when anticoagulation is used after hospital discharge. In general, undesirable outcomes outweigh desirable outcomes (Cuker et al., 2022). The CHEST guidelines also recommend that thromboprophylaxis is only recommended for hospitalized patients with COVID-19, not prolonged thromboprophylaxis after hospital discharge (Moores et al., 2020). Other guidelines listed in **Table 3** all state that post-discharge prophylaxis should be considered in terms of thrombotic risk and bleeding risk. Of course, these recommendations will be updated in light of changing evidence, but from the current evidence, the use of antithrombotic drugs after discharge requires caution.

**Table 3:** Recommendations of guidelines for thromboprophylaxis after discharge.

Guidelines	Suitable population for post-discharge anticoagulation	Recommendations for anticoagulation after discharge
ASH	Suspected or confirmed venous thrombus embolism (VTE) or other indication for anticoagulation	Outpatient anticoagulation prophylaxis is not used in discharged patients with COVID-19 without suspected or confirmed VTE or other indications for anticoagulation. Undesirable consequences may outweigh desirable consequences.
CHEST	Postdischargethrom boprophylaxis would result in net clinical benefit only if the risk of symptomatic VTE were found to be >1.8% within 35 to 42 days after release from the hospital.	Thromboprophylaxis is recommended only for hospitalized patients with COVID-19, rather than hospitalized patients plus prolonged thromboprophylaxis after discharge.
SSC-ISTH	COVID-19 hospitalized patients with high-risk VTE criteria, (including advanced age, ICU admission, cancer, previous VTE history, thrombophilia, severe inactivity, elevated d-dimer, or VTE improvement score ≥4).	Extended post-discharge thromboprophylaxis should be considered for all hospitalized patients with COVID-19 that meet high VTE risk criteria.
ACC	Patients at increased risk of VTE (including those with limited mobility and history of prior VTE or active malignancy).	After discharge, long-term prophylaxis with low-molecular-weight heparin or direct oral anticoagulants (DOACs) can reduce the risk of VTE but increase bleeding events, including major bleeding. While no data specific to COVID-19 exist, it is reasonable to employ individualized risk stratification for thrombotic and hemorrhagic risk, followed by consideration of extended prophylaxis (for up to 45 days) for patients with elevated risk of VTE.
ACF	Patients at increased risk of VTE (such as advanced age, cancer, obesity, pregnancy, congestive heart failure, or previous history of VTE).	Extended VTE prophylaxis is not necessary for all discharged COVID-19 patients. A multidisciplinary discussion at or near discharge is recommended to determine whether patients have persistent VTE risk factors, that prolonged post-hospital VTE prophylaxis may benefit, and to ensure access to VTE prophylaxis.
Belgian clinical guidance	Patients at increased risk of VTE (such as ICU admission, known thrombosis, obesity, high-dose estrogen use, immobilization, heart failure, respiratory failure, age 70 years, active cancer, personal or family history of VTE, and/or recent 3-month major surgery).	If other risk factors for VTE are present, it is recommended to extend thromboprophylaxis for 4 to 6 weeks after discharge.

ASH, American Society of Hematology; SCC, Scientific and Standardization Committee Communication; ACC, American College of Cardiology; ACF, Anticoagulation Forum; SCC-ISTH, Scientific and Standardization Committee of International Society of Thrombosis and Haemostasis.

## Antiplatelet In COVID

Platelets are at the forefront of COVID-19 pathogenesis, as they release a wide variety of molecules (including cytokines, alpha granules, dense granules and EVs) at different stages of the disease (Zaid et al., 2020; Rolla et al., 2021). Furthermore, COVID-19 patients had increased PS exposure in platelet extracellular vesicles (PEVs) (Rolla et al., 2021). Another study showed that SARS-CoV-2+ patients had higher counts of circulating platelet-derived extracellular vesicles (PLT-EVs) compared to healthy controls, with ROC curve analysis showing a sensitivity of 75% and specificity of 74% (Cappellano et al., 2021). SARS-CoV-2 can activate platelets and induce an inflammatory response that produces a wide range of immunomodulatory cytokines, chemokines, and other mediators. Endothelial injury promotes platelet activation, and in turn, chemotaxis of activated platelets recruits leukocytes, increases endothelial inflammation and thrombosis (Rolla et al., 2021). Recently, platelet activation inhibitors have garnered significant interest in COVID-19. A study suggests that antiplatelet therapy (including aspirin, clopidogrel, ticlopidine, prasugrel and ticagrelor) during COVID-19 hospitalization may be associated with a lower risk of death and shorter duration of mechanical ventilation without an increased risk of bleeding (Santoro et al., 2022). Aspirin is a mature drug with multiple effects such as inhibition of viral replication, anticoagulation, antiplatelet aggregation, anti-inflammatory and anti-lung injury (Mohamed-Hussein et al., 2020). Aspirin can inhibit prostaglandin E2 in macrophages and upregulate type I interferon to suppress viral replication. It can also reduce neutrophil aggregation and platelet activation. A study of covid-19 hospitalized patients showed that compared with patients who did not receive antiplatelet therapy, patients receiving aspirin had a significantly lower cumulative incidence of in-hospital death (Meizlish et al., 2021). Another study reported that tirofiban combined with aspirin and clopidogrel can effectively improve the ventilation/perfusion ratio in patients with severe respiratory failure due to COVID-19 (Viecca et al., 2020). There is very little data on combining antiplatelet and anticoagulant drugs in COVID-19. Though the dual mechanisms of antiplatelet and anticoagulation therapy on platelet thrombosis and hypercoagulability induced by COVID-19, may lead to synergistic effects (Matli et al., 2021). However, in hospitalized patients with moderate to severe COVID-19, anticoagulant heparin combined with aspirin may not be enough to inhibit thrombosis and increase bleeding risk (Rizk et al., 2021). There is also a lack of data on aspirin dosing and duration in COVID-19. In conclusion, aspirin can effectively inhibit inflammation, protect the endothelium, and prevent PS exposure after platelet activation. Based on pathophysiological insights, platelets may still represent a promising therapeutic target for COVID-19.

## Conclusions

Exploring the pathophysiological mechanism and impact of long COVID thrombosis will help improve understanding of early antithrombotic therapy and better prevent thrombotic sequelae. This article summarizes the effects of persistent viral replication, inflammation, hypoxia, and endothelial injury leading to thrombosis and organ disfunction in the long COVID. The procoagulant effects of EVs and PS exposure caused by injury to circulating blood cells and ECs are highlighted. Although the vaccine is an effective measure to prevent SARS-CoV-2 infection, there are still unmet medical needs. The risk of variants that evade vaccine immunity, vaccine contraindications, immunocompromised persons who respond poorly to vaccines, and the challenge of obtaining vaccines in some areas, result in many Covid-19 patients who need treatment. Research on the use of anticoagulants in early stage COVID-19 is rapid. Many experimental studies have shown that early antithrombosis reduces mortality and improves prognosis. In the future, early preventive antithrombotic therapy may be an important means to better solve COVID-19 sequelae.

## Author Contributions

CW conceived and wrote the first draft of the article. CY and HJ researched data for the article. XW and VN provided helpful comments and wrote the article. RX provided substantial contribution to discussion of content and wrote the article. JS designed the review, prepared the tables and figures, and wrote the manuscript. All authors read and approved the final manuscript.

## Funding

This work was supported by grants from the National Natural Science Foundation of China (81670659).

## Conflict of Interest

The authors declare that the research was conducted in the absence of any commercial or financial relationships that could be construed as a potential conflict of interest.

## Publisher’s Note

All claims expressed in this article are solely those of the authors and do not necessarily represent those of their affiliated organizations, or those of the publisher, the editors and the reviewers. Any product that may be evaluated in this article, or claim that may be made by its manufacturer, is not guaranteed or endorsed by the publisher.
